# Pediatric extranasal, EBV-negative, extranodal natural killer/T-cell lymphoma; case report

**DOI:** 10.3389/pore.2026.1612318

**Published:** 2026-06-24

**Authors:** Rachel Gallant, Osman Khan

**Affiliations:** Division of Pediatric Hematology-Oncology, Department of Pediatrics, University of Oklahoma Health Sciences Center, Oklahoma, OK, United States

**Keywords:** case report, EBV-negative, extranodal natural killer/T-cell lymphoma, natural killercell lymphoma, non-nasal, pediatric

## Abstract

Extranodal natural killer/T-cell lymphomas (ENKTCL) are rare, aggressive neoplasms primarily occurring in adults of Asian or Native American descent. Most are associated with Epstein-Barr virus (EBV) and originate in the nasopharyngeal region. We present the case of a fulminant, disseminated, non-nasal, EBV negative, seemingly de novo ENKTCL in an infant who presented with a five-day history of progressive abdominal distention, respiratory distress, irritability, non-bilious emesis, right eye proptosis, and pallor. Initial labs revealed leukocytosis, anemia and thrombocytopenia. Computerized axial tomography revealed densities in the right optic nerve, superior orbits, thymus, posterior mediastinum, pericardium, myocardium, axillary lymph nodes, adrenal glands, kidneys and pancreas. The patient rapidly deteriorated, and despite extensive resuscitation, ultimately died. Autopsy revealed tumors in many organs, displaying vascular damage and tissue necrosis with lymphoid infiltration. The likely diagnosis of EBV-negative ENKTCL was confirmed after further histologic analysis. Pediatric ENKTCL is a particularly uncommon tumor, especially given our patient's very young age and EBV-negative status. Determining the best treatment regimen for these children is challenging due to limited data. Treatment typically consists of combination chemotherapy and radiation, but targeted therapies are being explored with promising results.

## Introduction

Natural killer (NK) cell lymphomas are a rare malignancy in both adults and children with an incidence of about 0.65 per 100,000 in the United States [1]. A subset of these, extranodal NK-/T-cell lymphoma (ENKTCL), occurs most commonly in people of Asian and Pacific Island descent, and rarely in the Western Hemisphere [1, 2]. ENKTCL typically presents in the nasopharynx, and almost all are Epstein-Barr virus (EBV)-positive. These tumors are also highly aggressive and confer a poor prognosis with a 5-year overall survival rate of approximately 70% in lower stage disease and only 50% in advanced disease [3]. ENKTCL in children is particularly rare. Here, we present the case of a very rare infantile, extranasal, EBV-negative, ENKTCL in a previously healthy infant. The diagnosis was made post-mortem due to the fulminant nature of the clinical course. The presentation in infancy, the site of disease, and the EBV-negativity all render the case most unusual.

## Case description

**FIGURE 1:**
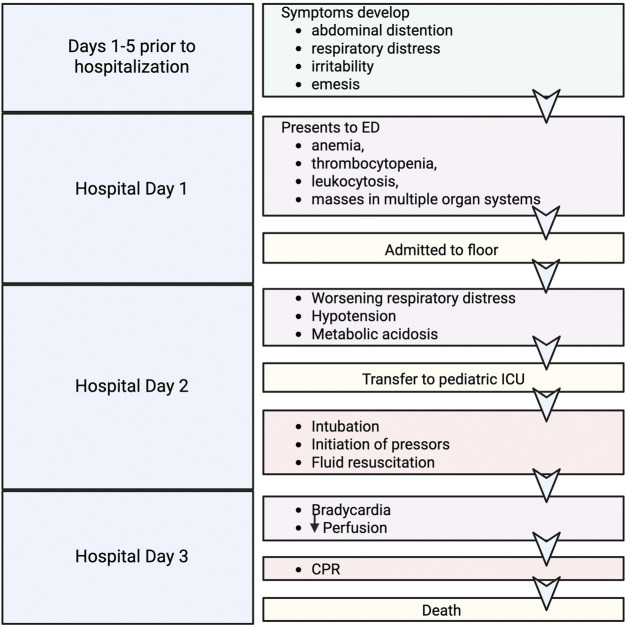
Timeline of clinical course. Abbreviations: CPR: cardiopulmonary resuscitation. Created with Biorender.

A previously healthy infant presented with a 5-day history of progressive abdominal distention, respiratory distress, irritability and occasional, non-bilious emesis (Figure 1). On physical exam, the patient was afebrile, tachycardic, tachypneic, pale, and in moderate respiratory distress with grunting and subcostal retractions. Proptosis of the right eye was prominent, with a yellow-green discoloration of the eyelid. The abdomen was markedly distended, and eczematous dermatitis of the scalp was present but no other notable skin lesions. The remainder of the physical exam was unremarkable.

Laboratory work-up revealed a white blood cell count of 29.4 K/uL with 59% neutrophils and 38% lymphocytes, hemoglobin of 7.3 g/dL, and platelet count of 128 K/uL. A plain chest radiograph revealed a left paraspinal density projecting into the retrocardiac region with a vague opacity in the right upper lobe. Computer axial tomography (CT) revealed a right retroocular density along the optic nerve and superior orbits. CT imaging of the chest revealed a 2.5 cm low attenuation thymic mass, a 4.0 cm left paraspinal mass in the posterior mediastinum, and a separate 2.1 cm elliptical soft tissue mass anterior to the paraspinal mass at the base of the left lung lower lobe. Multifocal pericardial and myocardial involvement were noted at the right ventricular outflow tract and surrounding the left ventricle. There were conglomerated nodes in the left axillary region, the largest measuring 1.8 cm. The abdominal CT scan revealed masses of similar attenuation totally replacing both adrenal glands in addition to involvement of both kidneys and the pancreas.

Shortly after admission, the patient’s clinical status rapidly deteriorated and required transfer to the pediatric intensive care unit, intubation, mechanical ventilation, and hemodynamic support. A profound metabolic acidosis was identified and treated with fluid resuscitation including packed red blood cell transfusion, sodium bicarbonate, and vasoactive infusions. However, after initial clinical improvement, the patient acutely deteriorated becoming bradycardic with marked deterioration in perfusion despite adequate oxygenation. Cardiopulmonary resuscitation was initiated, but despite these efforts, the cardiac rhythm deteriorated to asystole with no palpable pulses or other evidence of perfusion, and the patient was pronounced dead. After obtaining consent, an unrestricted autopsy was performed.

## Diagnostic assessment

**FIGURE 2:**
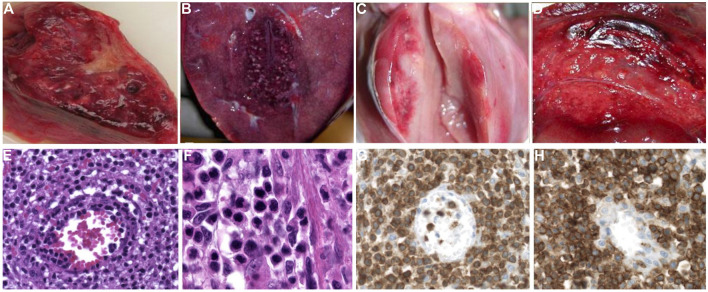
Gross and microscopic findings. Tumor nodules in the thymus **(A)**, lung **(B)**, heart **(C)** and adrenal glands **(D)**. Microscopic evaluation of the lymph nodes revealing diffuse and infiltrative lymphoid proliferation with an angiocentric predilection **(E,F)** and positive immunohistochemical stains for cytoplasmic CD3 **(G)** and CD56 **(H)**.

Post-mortem examination revealed multiple ill-defined and hemorrhagic tumor nodules involving many organs including the thymus, heart, lungs, liver, pancreas, spleen, small intestines, adrenal glands and kidneys (Figure 2). No primary site of origin could be identified.

Microscopic examination of each organ demonstrated extensive replacement by a diffuse and infiltrative lymphoid proliferation. Tumor cells were monotonous, small to medium sized cells with clear cytoplasm and round to irregular nuclei and inconspicuous nucleoli (Figure 2). Tumor cells displayed an angiocentric and angiodestructive predilection with resulting prominent areas of tissue necrosis and vascular damage. In the adjacent necrotic tissue regions, a marked number of apoptotic bodies were identified. Immunocytochemical staining of the tumor was positive for CD56, cytoplasmic CD3 (cCD3), TIA-1, and granzyme-B (Figure 2). Immunoperoxidase staining for lysozyme, myeloperoxidase, CD57, CD79a, and CD117 was negative. These findings, in conjunction with negative polymerase chain reaction testing for a T-cell receptor gene mutation, were suggestive of a diagnosis of an ENKTCL. However, *in situ* hybridization (ISH) for EBV was negative.

## Discussion

Malignancies arising from NK-cells are rare neoplasms, with primarily case series and individual case reports presented in the literature. Our case is particularly unusual in its presentation due to the young age, non-nasal distribution, and EBV-negativity as well as for its widespread dissemination and rapidly fatal course.

The classification of NK-cell lineage tumors has evolved in recent years. The current WHO classification (5^th^ edition) divides T- and NK-cell neoplasms into leukemias, primary cutaneous T-cell lymphomas, intestinal lymphoid proliferations and lymphomas, hepatosplenic lymphoma, anaplastic large cell lymphoma, T-follicular helper lymphoma, EBV-positive T-cell and NK-cell lymphomas, and EBV-positive T- and NK-cell lymphoid proliferations and lymphomas of childhood [4]. EBV-positive T-cell and NK-cell lymphomas are divided into: (1) extranodal NK/T-cell lymphoma (ENKTCL, previously extranodal NK/T-cell lymphoma, nasal-type) and (2) EBV-positive nodal T- and NK-cell lymphoma (Table 1) [4]. Other EBV-positive T- and NK-cell lymphoid proliferations and lymphomas of childhood include: (1) Severe mosquito bite allergy (2) Hydroa vacciniforme lymphoproliferative disorder (3) systemic chronic active EBV disease, and (4) systemic EBV-positive T-cell lymphoma of childhood [4].

**TABLE 1:** Immunohistochemical profile of NK/T-cell Lymphoma (ENKTCL). Comparison of immunohistochemical profile of ENKTCL and the patient case. NA: not available.

Immunohistochemical marker	ENKTCL	Patient case
CD56	Positive	Positive
CD2	Positive	*NA*
Surface CD3	Negative	Negative
Cytoplasmic CD3	Positive	Positive
Cytotoxic molecules (perforin, granzyme B, TIA-1)	Positive	Positive
T-cell receptor rearrangement	Negative	Negative
Other T-cell markers (CD4, CD5, CD7, CD9)	Negative	*NA*
Epstein-Barr virus	Positive (usually)	Negative

ENKTCLs demonstrate great morphologic diversity but typically arise in the aerodigestive tract and therefore were previously classified as ENKTCL, nasal type. Presenting symptoms often include nasal obstruction, epistaxis and eventually midfacial destruction. Metastatic disease is rare, but ENKTCL most often spreads to skin, testes, gastrointestinal tract, lymph nodes and infrequently bone marrow [5, 6]. Though the aerodigestive tract is the most common site, extranodal primary disease has been reported without nasal involvement, prompting the change in nomenclature to ENKCTL [7]. The predominant sites of primary extranasal disease are the skin, testes, soft tissue, gastrointestinal tract and spleen [6, 8].

The histology of ENKCTLs is characterized by a broad cytological spectrum and can be difficult to diagnose as tumors may be embedded in large necrotic areas with neoplastic infiltrates demonstrating minimal cytologic atypia admixed with inflammatory cells. ENKTCL most commonly arises from NK-cells, although it can also arise from CD8^+^ T-cells in a subset of cases. ENKTCL cells display intense positivity for CD56 antigen, however, often lack other mature NK-cell and T-cell markers such as CD57, CD16, CD4, and CD8 [7]. Although these cells lack surface antigen CD3, they do express cytoplasmic CD3 [9, 10]. They also express cytotoxic molecules including granzyme B, TIA-1, GMP17, and perforin and typically clonal rearrangement of both T-cell receptor (TCR) and immunoglobulin heavy chain genes are absent [6]. In contemporary ENKTCL WHO classification, EBV positivity is a required diagnostic criterion. It is identified by *in situ* hybridization in almost all cases of nasal ENKTCL especially in patients of Asian descent, but historically EBV-positivity is less consistent in extranasal disease and in the Caucasian population [6].

There are some important limitations in the diagnosis of ENKTCL in our case presentation. First, our case was negative for EBV by ISH but given that it was performed on post-mortem tissue, RNA degradation could have contributed to the negative result. Furthermore, it is difficult to definitively distinguish between cytoplasmic and surface expression of CD3 without flow cytometry. Though flow cytometry was not performed in this case, there appears to be cytoplasmic staining of CD3 on IHC. Therefore, we must consider other CD3- and CD56-positive neoplasms in the differential diagnosis. Aggressive NK-cell leukemia expresses cytoplasmic CD3, surface CD56, and cytotoxic molecules as is seen in our case [4, 11]. Though the clinical presentation of NK-cell leukemia overlaps with our patient in some aspects including fevers, diffuse lymphadenopathy, and hepatosplenomegaly, typically there is leukemic involvement of which there was no evidence at diagnosis in our case. Large granular lymphocytic leukemias (NK-cell or T-cell type) also express cytotoxic molecules, CD3, and CD56, however the clinical course is typically indolent and can even be asymptomatic in 1/3 of cases which is strikingly different from the acute fulminant course described in our patient [4, 11]. Furthermore, TCR gene rearrangement is typically positive in T-large granular lymphocytic leukemia but was negative in our case. EBV-positive nodal T- and NK-cell lymphoma expresses cytotoxic markers and CD3, but differs from our case as only a minor subset of cases express CD56 [4, 11]. Furthermore, EBV must be positive to make the diagnosis. Hepatosplenic T-cell lymphoma, anaplastic large cell lymphoma, and peripheral T-cell lymphoma can express CD3, CD56, and cytotoxic markers, but TCR gene rearrangement is positive in these types of lymphoma and the clinical course is quite distinct from our case [4, 11]. T-lymphoblastic leukemia (T-ALL) expresses CD3, but most have clonal TCR rearrangements and only a minority express CD56 [4, 11]. Peripheral leukemic blasts or cytopenias would be expected to be more prominent in T-ALL as well. Finally, blastic plasmacytoid dendritic cell neoplasm shares features of our case with CD56 positivity, absence of TCR gene rearrangement and EBV, but differs in that CD3 is typically negative [4, 11]. Though our case does not fit perfectly with the diagnostic criteria for any of the previously mentioned NK or T-cell neoplasms, the histologic clinical features fit best with ENKTCL especially considering that EBV may have been negative secondary to RNA degradation in the post-mortem tissue. As such, ENKTCL was the final diagnosis of this patient.

The genetic and molecular landscape of ENKCTL is still being explored, but some recurrent genetic alterations have been identified. Loss of heterozygosity at chromosomes 6q, 11q, 13q, and 17p have been reported [7], and notably, deletions of the 6q21-23 region result in the loss of various tumor suppressor genes [3]. Some genetic alterations vary by geographic location. For instance, *TP53* mutations have been reported primarily in Asian cohorts [3, 7]. Other genetic variations seem to differ by the cell of origin with *STAT3*, *DDX3X*, *KMT2C*, *JAK2*, *KMT2D*, *EP300*, *STAT5B*, and *STAT5A* being more common in NK-cell derived ENKTCL and *EPHA1*, *TP53*, *ARID1A*, *PTPRQ*, *NCOR2*, *PPFIA2*, *BCOR*, *PTPRK*, and *HDAC* being more common in T-cell derived ENKTCL [7, 12]. The genes affected in NK-cell derived ENKTCL tend to result in upregulation of the JAK/STAT pathway while mutations in T-cell derived ENKTCL result in upregulation of the RAS-MAPK pathway and epigenetic modification [7, 12]. However, these mutations exhibit a much lower mutational burden than in other types of non-Hodgkin lymphoma, suggesting that there is another driver of oncogenesis, possibly EBV [7]. Furthermore, the genetic and molecular profile of pediatric ENKTCL may differ from adult tumors with mutations in *KMT2C*, *MST1*, *HLA-A*, and *BCL11A* being recurrently reported [13].

Pediatric ENKTCL is quite uncommon. An international database review revealed that only 3% (n = 26) of ENKCTL cases occurred in patients <20 years of age highlighting its rarity in pediatrics [14]. Although histologically adult and pediatric ENKTCL are similar, there are some differences in their clinical presentation and outcomes. Nearly all cases of adult nasal ENKTCL and up to 94% of extranasal ENKTCL are associated with EBV infection [8, 15]. In pediatric nasal ENKTCL, EBV is also typically positive, however, extranasal disease lacks definitive EBV status [14, 15]. Although the prognosis in adults is historically poor [8], combination chemotherapy and radiation has improved outcomes with survival rates up to 70%–80% in localized disease (Stage I/II) and 50% in advance disease (Stage III/IV) [3]. Outcomes for pediatric patients is highly variable depending on the literature source ranging from 30% to 80%, but similarly notes poorer survival with higher stage disease [1, 16, 17].

Given the rarity of this type of lymphoma, all reported clinical studies pertaining to treatment are retrospective patient series or case reports. Clinical studies regarding non-nasal extranodal disease are even more rare, with a typically aggressive course [14]. One of the largest cohorts of pediatric patients with ENKTCL lymphoma (n = 34) demonstrates much poorer overall survival in those with Stage III/IV disease than those with Stage I/II (26.0% and 66.2% respectively) [18]. Furthermore, those with extranasal ENKTCL lymphoma had lower event free and overall survival compared to those with nasal disease (EFS: 33.3% vs. 54.2%, OS: 62.5% vs. 75.1%) although this did not reach statistical significance [18]. Chan et al reported 34 cases of extranodal ENKTCL with poor response to multi-agent chemotherapy [8]. Among 29 patients with follow-up information, 24 (83%) died in 1 week to 3 years from diagnosis (median survival 3.5 months), 3 (10%) were alive with relapsed disease (relapse at 5 months, 2 years, and 2.5 years), and 2 (7%) remained in remission at 3 and 5 years [8]. Wang et al reported better outcomes in a case series of 22 pediatric patients and found that those who received treatment with chemotherapy alone, radiation alone, or combination chemotherapy all had an overall survival of approximately 63% [13]. Most of these patients (>70%) were advanced stage, and likely due to the small sample size, they noted no difference in any clinical features and prognosis [13]. There seems to be a high rate of chemotherapy resistance thought to be associated with the expression of the multi-drug resistance (MDR) 1 gene as well as poor drug delivery because of significant tissue necrosis which may contribute to poor response to therapy [6, 14, 19]. Typical therapy for limited stage disease (I/II) includes combination of chemotherapy and radiation therapy. PEG-asparaginase containing regimens are generally recommended for those with advanced disease (stage III/IV) and have led to improved survival [14, 18, 20].

For those with advanced or relapsed/refractory disease, hematopoietic stem cell transplant (HSCT) has previously been reported to have promising results [16–18, 21–23]; however, survival rates after transplant are highly variable and dependent upon tumor burden at the time of transplant and the geographic region [24]. Yokoyama et al presented a case of stage III nasal, extranodal NK-cell lymphoma that relapsed after initial chemotherapy and achieved sustained remission (33 months post-HSCT) after unrelated cord blood transplant (UCBT) [22]. Yoshimasu et al reported an adolescent male with stage IV blastoid NK-cell lymphoma who failed autologous transplant but was successfully treated with UCBT [23]. Nawa et al, likewise presented the case of an adolescent female who underwent allogeneic transplant who remained in remission at 30 months post-transplant [21]. Suzuki et al reviewed 22 patients (adult and pediatric) with ENKTCL who were treated with HSCT and found that those who underwent HSCT had better overall survival than those treated with chemotherapy alone (approximately 55% and 30% respectively) [25]. There was no statistically significant difference in outcomes in those who underwent allogeneic versus autologous transplant likely due to the small sample size. However, they noted a significantly improved survival in those who achieved complete remission prior to transplant compared to those with active disease [25].

Despite combination chemotherapy and radiation, there is still significant room for improvement in treatment and outcomes of ENKTCL prompting investigation of additional therapies. PD-L1 inhibitors such as pembrolizumab and nivolumab have been successful in the treatment of ENKTCL. Interestingly, PD-L1 inhibitors seem to be effective independent of the level of PD-L1 expression. Due to CD30 and CD38 expression, brentuximab and daratumumab have been investigated, but have not yet shown convincing results [7, 26]. As the JAK/STAT pathway has been found to be recurrently upregulated in ENKTCL tumor cells, JAK inhibitors are being investigated and have demonstrated efficacy *in vitro* [7, 27–29]. Use of ruxolitinib (a JAK inhibitor) in combination with chemotherapy induced remission *in vivo* in a single case report [29]. A subset of ENKTCL tumors have been found to upregulate epigenetic modification and the RAS pathway prompting investigation of HDAC inhibitors and mTOR inhibitors [7, 30]. *In vitro* success with mTOR inhibitors has been demonstrated particularly in EBV-positive ENKTCL tumor cell lines [30]. Although HDAC inhibitors have not been found to be effective as monotherapy, they are being further studied in combination with chemotherapy [7].

Pediatric ENKTCL is a particularly uncommon tumor, with our patient representing an extremely unusual subset given the patient’s age and EBV-negative status. Determining the best treatment regimen for these children is challenging given limited data. Typically, therapy regimens are extrapolated from the published adult data but dedicated pediatric studies are required to truly understand the efficacy and outcomes in children. Promising targeted therapies for pediatric ENKTCL are on the horizon, but there is still much to learn about the biology, immunology, natural history, and response to treatment in ENKTCL particularly in pediatrics.

## Data Availability

The original contributions presented in the study are included in the article/supplementary material, further inquiries can be directed to the corresponding author.
